# Spare Parts: Repurposing Tissue in Facial Feminization Surgery

**DOI:** 10.1007/s00266-026-05642-4

**Published:** 2026-01-20

**Authors:** Sumun Khetpal, Anne E. Hall, Kaavian Shariati, Nghiem H. Nguyen, Alex A. Argame, Julissa Molina-Vega, Jake Alford, Abie H. Mendelsohn, Justine C. Lee

**Affiliations:** 1 Division of Plastic and Reconstructive Surgery, University of California, 200 Medical Plaza, Suite 460, Los Angeles, CA 90095 USA; 2 Los Angeles Center for Ear, Nose, Throat and Allergy, Los Angeles, CA USA

**Keywords:** Facial feminization surgery, Rhinoplasty, Genioplasty, Lip augmentation, Split calvarial bone graft, Dermal graft

## Abstract

Single-stage facial feminization surgery (FFS) offers a unique opportunity for intraoperative access to nearly every tissue type used for grafting in plastic surgery, opening the possibilities for creative repurposing of autologous tissues. In this work, we describe methods of utilizing calvarial bone, mandibular bone, thyroid cartilage, and dermal fat harvested from routinely accessed sites or typically excised and discarded during FFS. We summarize the usage of split calvarial bone from the parietal region and osteotomized gonial angles as grafts for the osteotomy sites of the anterior table of the frontal sinus and osseous genioplasty and as a septal graft in rhinoplasty. We also describe the repurposing of thyroid cartilage from the chondrolaryngoplasty as shield grafts for rhinoplasty. Lastly, we described the use of dermal fat grafts from excised, de-epithelialized forehead and hair-bearing scalp for lip augmentation and as a radix graft. These techniques highlight the availability of autologous tissue throughout the face and deliberate intraoperative sequencing when encountering the potential need for tissue supplementation. Our multi-year experience has shown these methods to be reliable and well-received by patients. As such, the current work presents the surgical technique and intraoperative usage of “spare parts” within single-stage FFS.

*Level of Evidence IV* This journal requires that authors assign a level of evidence to each article. For a full description of these Evidence-Based Medicine ratings, please refer to the Table of Contents or the online Instructions to Authors www.springer.com/00266.

**Supplementary Information:**

The online version contains supplementary material available at 10.1007/s00266-026-05642-4.

## Innovation and Technique Description

Comprehensive access to craniofacial skeletal, cartilaginous, and soft tissue structures is a hallmark of one-stage facial feminization surgery (FFS) [1–8]. As such, the opportunity exists to harvest autologous tissue without additional donor site morbidity and repurpose discarded tissues as graft materials. These techniques have been utilized over several years in a high-volume, single-stage FFS practice, informing the operative methods we describe here. We highlight some of these uses from our multi-year FFS experience.

*Bone* For any osteotomy, the best bone healing occurs in the acute period. As such, every effort should be made ensure union. While bone dust from frontal bone recontouring is widely re-used as bone spackle in nearly all cranioplasty procedures, it is insufficient for wider gaps between osteotomized bone. Different from split calvarium incorporating the entirety of the outer cortex, we obtain split outer cortical bone from the parietal region as thin pieces to bridge gaps between the anterior table and native frontal bone in every Type III or Type I+ [9] forehead procedure. Harvest occurs approximately 1 cm behind the coronal suture and 1 cm away from the sagittal suture using a stubby Tessier osteotome, avoiding the diploic space as much as possible. These thin pieces are secured with titanium plates and screws with the larger anterior table piece to the native bone (Supplementary Digital Content 1). Similarly, we have used mandibular bone from the gonial angle osteotomies for the same purpose, albeit more shaping is required to reduce the thickness of resected mandible.

We have also found that split outer cortical parietal bone, bone retrieved from the gonial angles, and the resected central piece from two-piece osseous genioplasties to be useful as bone grafts to the horizontal osteotomy of the osseous genioplasty, particularly in the setting of correcting cant or lengthening genioplasty. Additionally, the central wedge resected in two-piece osseous genioplasties is useful when secured to the inferior edge of the chin, particularly for cleft chin correction, as we have previously described [6].

**Fig. 1:**
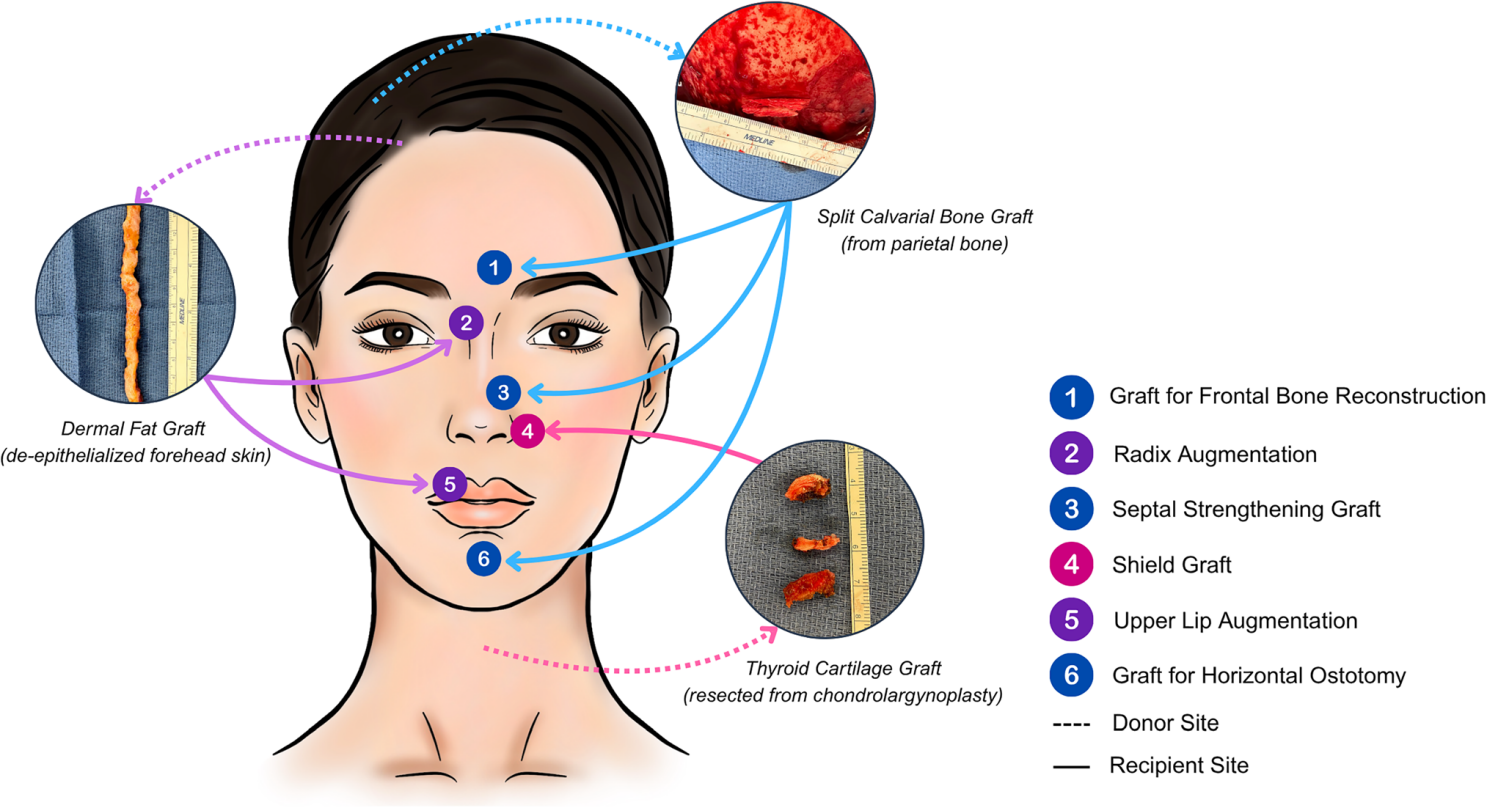
Diagram illustrating intraoperative autologous tissue repurposing of bone, cartilage and soft tissue in FFS. Tissues from donor sites (indicated by blue arrows) are redirected to recipient sites (indicated by red arrows) across multiple facial subunits to support structural reconstruction and esthetic goals

In settings where septal cartilage is weak, calvarial bone grafts and ethmoid bone serve as strong septal grafts, offering a thin and strong alternative to either autologous or cadaveric costal cartilage grafts (Fig. 1). However, we have strictly only used these grafts at or below the anterior septal angle, not as classic septal extension grafts, given the stiffness of bone.

*Cartilage* Thyroid cartilage is an unconventional source of cartilage that can only be found during this combined operation. In open incisional chondrolaryngoplasty approaches that access the thyroid transcervically, it is often routine to harvest an en bloc cartilage strip measuring approximately 5 mm × 20 mm, depending on body height. However, in our typical transvestibular endoscopic chondrolaryngoplasty [10, 11], the resected cartilage is usually obtained in a more piecemeal fashion having insufficient length as septal extension, spreader, or alar grafts. However, we have found these thyroid cartilage specimens to be a useful source for shield grafts.

*Soft tissue* Dermal fat graft harvested either from the forehead for hairline advancement or a galeal fat graft harvested from excised excess scalp in coronal approaches are valuable for soft tissue augmentation. We have used these tissues for augmentation of the upper lip, radix, and nasal dorsum. Across our multi-year experience, these grafts have consistently provided reliable intraoperative volume enhancement without additional donor site morbidity. For the upper lip, two small stab incisions at the commissures are made to tunnel a 14- or 16-French introducer from one oral commissure to the other. The dermal fat graft is then delivered and provides a durable, natural volume enhancement.

**Fig. 2:**
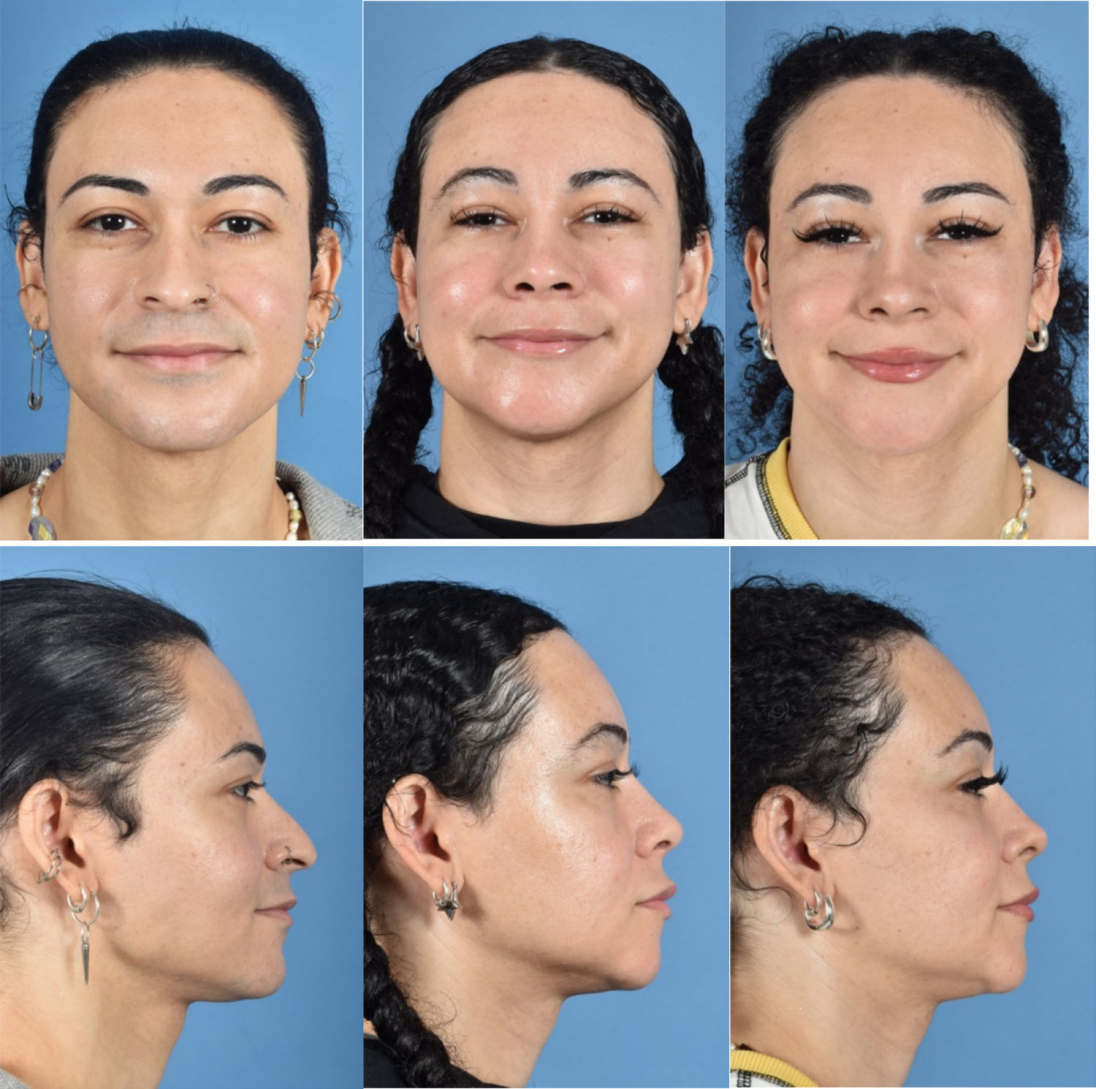
Preoperative photographs (*first column*), 5-month postoperative photographs (*second column*), and 1-year postoperative photographs (*third column*) of a patient who underwent single-stage full FFS. Key procedures performed included 2-mm frontal sinus setback (Type 1+), coronal brow lift, reduction genioplasty, gonial angle reduction, fat grafting to the temple and malar regions, dermal fat graft to the upper lip, open septorhinoplasty (dorsal preservation style) with septal extension graft, tip refinement sutures, osteotomies, inferior turbinate reduction, and dermal fat graft to the radix. The procedures combined resulted in an overall enhanced feminine appearance

The same soft tissues are particularly useful in ethnic rhinoplasties. In individuals of African, Asian, and certain Latino descent, radix and dorsal augmentation may be necessary to achieve a more balanced nasal profile as well as a narrowing of the nose. To minimize displacement, we typically use a limited dissection and a shortened 16-French introducer to help guide the graft to the glabella. The graft is then secured percutaneously over a pledget at the superior aspect and to the septum inferiorly (Fig. 2).

## Discussion

In bony reconstruction, the best opportunity for union is the first. For frontal bone reconstruction, the long-term sequelae of inadequate attention to maximizing union include: 1. Bony deformities that become obvious over time as skin thins; or 2. Areas of nonunion that are unmasked during sinus infections. For osseous genioplasties, the addition of readily available bone grafting clearly contributes to union and long-term stability of osteotomies.

In soft tissue augmentation, we have found that standard fat grafting to the lips is less durable and predictable than the forehead dermal fat graft or the scalp galeal fat graft. Although the initial swelling is substantial, swelling reduces by three months after surgery with a final augmentation result that is natural and subtle. We have encountered no circumstances in which a patient has requested reduction or removal of the dermal fat graft postoperatively.

While the literature has reported the usage of dermal fat grafts from scars or the gluteal fold for dorsal augmentation [12], this is the first report in which the forehead and scalp have been used. We have predominantly used this technique for ethnic rhinoplasties where the radix and/or dorsum are deficient. However, we have found this technique to be occasionally helpful should a contour abnormality of the dorsum arise, albeit contour abnormalities have substantially decreased with the conversion from structural to preservation rhinoplasties in the senior author’s practice.

For optimal usage of the available autologous tissues, some degree of intentional procedural sequencing must occur. Our general procedure is to start with the frontal bone reconstruction to have sufficient split calvarial bone and soft tissue available in anticipation for usage lower in the face. This is followed by the intraoral procedures including the transvestibular endoscopic chondrolaryngoplasty. In rare occasions when the defects of the frontal bone require a substantial amount of bone, the gonial angles resection has preceded closure of the frontal bone reconstruction. The rhinoplasty and soft tissue work are always sequenced last to benefit from any available donor tissues.

Further opportunities for innovation are likely to exist. For example, we have only used thyroid cartilage as a shield graft due to our minimally invasive approach for the chondrolaryngoplasty. However, maximally sparing cuts during this advanced endoscopic technique might be called for to continue to advance these goals. Such possibilities highlight the ongoing potential for creative, experienced-based solutions using tissue resources in FFS.

## Supplementary Information

Below is the link to the electronic supplementary material.

Supplementary file1 (MP4 289671 KB) This video illustrates the operative techniques featured in this paper. We demonstrate our process of harvesting split calvarial bone graft to be used in promoting union in anterior table and mandibular reshaping and for septal strengthening in open septorhinoplasty. We also describe our process of harvesting dermal fat grafts from the forehead to be repurposed to the radix and the upper lip. Lastly, we show our method of harvesting thyroid cartilage through a minimally invasive approach.
